# The One Health Concept: 10 Years Old and a Long Road Ahead

**DOI:** 10.3389/fvets.2018.00014

**Published:** 2018-02-12

**Authors:** Delphine Destoumieux-Garzón, Patrick Mavingui, Gilles Boetsch, Jérôme Boissier, Frédéric Darriet, Priscilla Duboz, Clémentine Fritsch, Patrick Giraudoux, Frédérique Le Roux, Serge Morand, Christine Paillard, Dominique Pontier, Cédric Sueur, Yann Voituron

**Affiliations:** ^1^CNRS, Interactions Hôtes-Pathogènes-Environnements (IHPE), UMR5244, Université de Perpignan Via Domitia, Université de Montpellier, Ifremer, Montpellier, France; ^2^Université de La Reunion, UMR PIMIT (Processus Infectieux en Milieu Insulaire Tropical), INSERM 1187, CNRS 9192, IRD 249, Sainte-Clotilde, La Réunion, France; ^3^UMR Ecologie Microbienne, CNRS, INRA, VetAgro Sup, Claude Bernard University Lyon 1, Université de Lyon, Villeurbanne, France; ^4^UMI 3189 “Environnement, Santé, Sociétés”, Faculty of Medicine, Cheikh Anta Diop University, Dakar-Fann, Senegal; ^5^Téssékéré International Human-Environment Observatory Labex DRIIM, CNRS and Cheikh Anta Diop University, Dakar, Senegal; ^6^Université de Perpignan Via Domitia, Interactions Hôtes-Pathogènes-Environnements (IHPE), UMR5244, CNRS, Ifremer, Université de Montpellier, Perpignan, France; ^7^Institut de Recherche pour le Développement, Maladies Infectieuses et Vecteurs, Ecologie, Génétique, Evolution et Contrôle (MIVEGEC), IRD, CNRS, Université de Montpellier, Montpellier, France; ^8^Laboratoire Chrono-Environnement, UMR 6249 CNRS/Université Bourgogne Franche-Comté Usc, INRA, Besançon, France; ^9^Institut Universitaire de France, Paris, France; ^10^Ifremer, Unité Physiologie Fonctionnelle des Organismes Marins, Plouzané, France; ^11^Institut des Sciences de l’Évolution (ISEM), UMR 5554, CNRS, Université de Montpellier, CIRAD, IRD, EPHE, Montpellier, France; ^12^UPR ASTRE, CIRAD, Montpellier, France; ^13^Laboratoire des Sciences de l’Environnement Marin (LEMAR), Institut Universitaire Européen de la Mer, Université de Bretagne Occidentale, UMR 6539, CNRS, UBO, IRD, Ifremer, Plouzané, France; ^14^Laboratoire de Biométrie et Biologie Evolutive UMR5558, CNRS, Université de Lyon, Université Claude Bernard Lyon 1, Villeurbanne, France; ^15^LabEx Ecofect, Eco-Evolutionary Dynamics of Infectious Diseases, University of Lyon, Lyon, France; ^16^Université de Strasbourg, CNRS, IPHC, UMR 7178, Strasbourg, France; ^17^Laboratoire d’Ecologie des Hydrosystèmes Naturels et Anthropisés, UMR 5023, CNRS, Université Claude Bernard Lyon1, Université de Lyon, Villeurbanne, France

**Keywords:** One health, EcoHealth, infectious disease, non-communicable disease, multifactorial disease, ecotoxicology, interdisciplinary research, public health

## Abstract

Over the past decade, a significant increase in the circulation of infectious agents was observed. With the spread and emergence of epizootics, zoonoses, and epidemics, the risks of pandemics became more and more critical. Human and animal health has also been threatened by antimicrobial resistance, environmental pollution, and the development of multifactorial and chronic diseases. This highlighted the increasing globalization of health risks and the importance of the human–animal–ecosystem interface in the evolution and emergence of pathogens. A better knowledge of causes and consequences of certain human activities, lifestyles, and behaviors in ecosystems is crucial for a rigorous interpretation of disease dynamics and to drive public policies. As a global good, health security must be understood on a global scale and from a global and crosscutting perspective, integrating human health, animal health, plant health, ecosystems health, and biodiversity. In this study, we discuss how crucial it is to consider ecological, evolutionary, and environmental sciences in understanding the emergence and re-emergence of infectious diseases and in facing the challenges of antimicrobial resistance. We also discuss the application of the “One Health” concept to non-communicable chronic diseases linked to exposure to multiple stresses, including toxic stress, and new lifestyles. Finally, we draw up a list of barriers that need removing and the ambitions that we must nurture for the effective application of the “One Health” concept. We conclude that the success of this One Health concept now requires breaking down the interdisciplinary barriers that still separate human and veterinary medicine from ecological, evolutionary, and environmental sciences. The development of integrative approaches should be promoted by linking the study of factors underlying stress responses to their consequences on ecosystem functioning and evolution. This knowledge is required for the development of novel control strategies inspired by environmental mechanisms leading to desired equilibrium and dynamics in healthy ecosystems and must provide in the near future a framework for more integrated operational initiatives.

## Introduction

**Figure 1:**
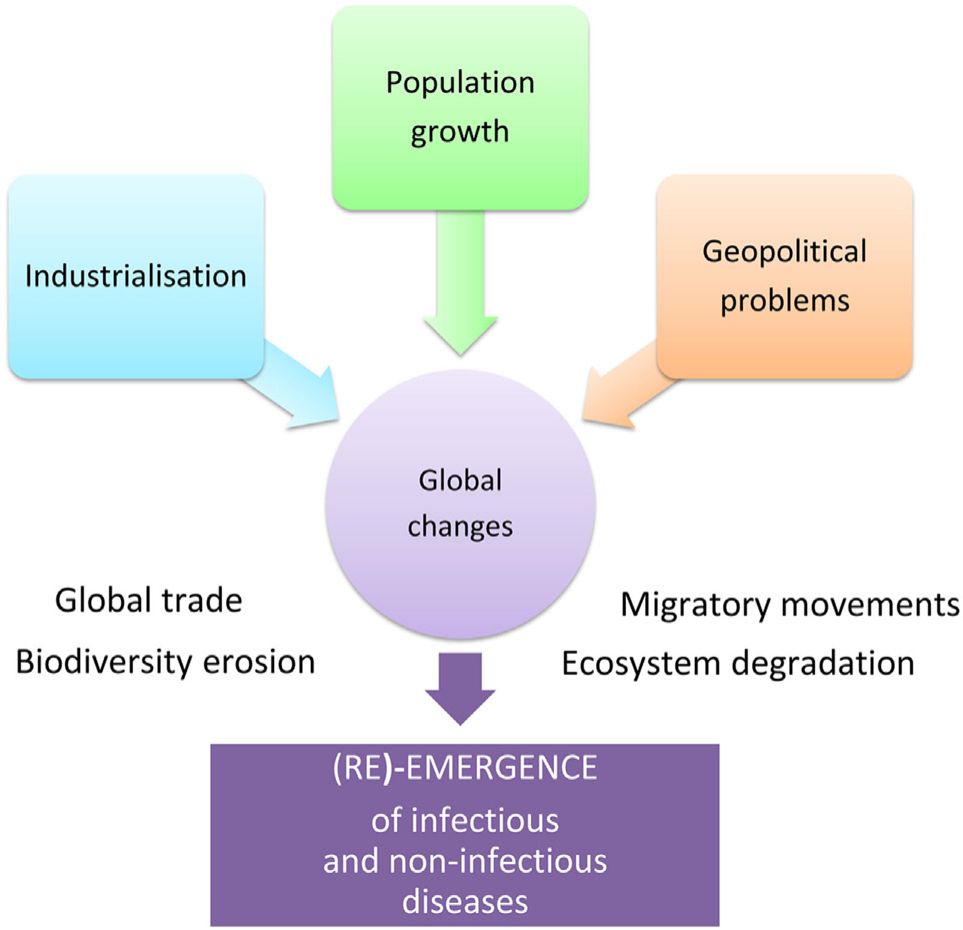
Global changes favor the (re)-emergence of infectious and non-infectious diseases.

**Figure 2:**
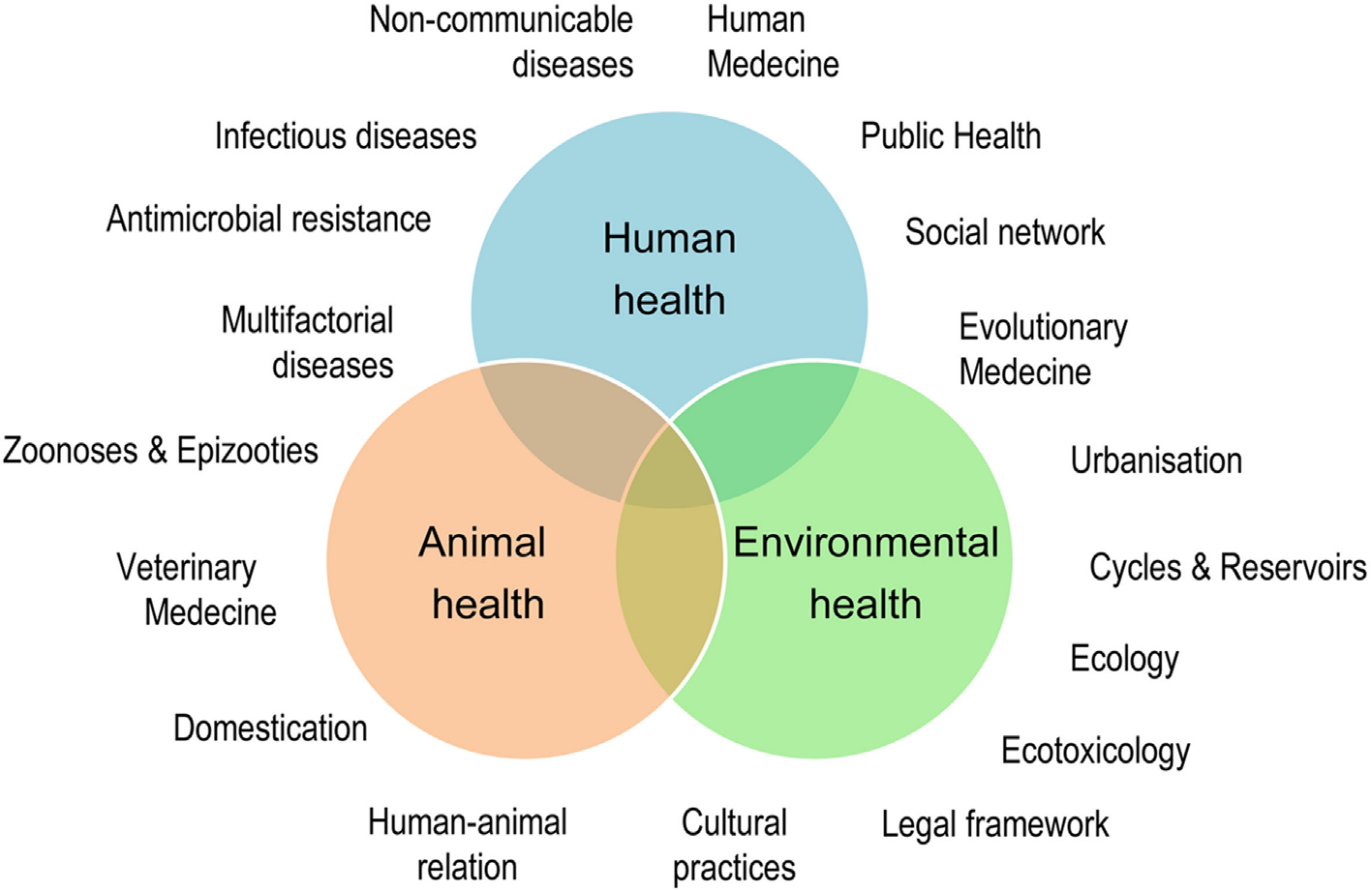
The One Health concept: a holistic, transdisciplinary, and multisectoral approach of Health.

Human population increase, industrialization, and geopolitical problems accelerate global changes causing significant damage to biodiversity, extensive deterioration of ecosystems, and considerable migratory movement of both mankind and species in general. These rapid environmental changes are linked to the emergence and re-emergence of infectious and non-infectious diseases (Figure 1). Over recent years, certain zoonoses, such as bird flu or the Ebola and Zika viral epidemics, have illustrated this fact to the whole world demonstrating the interdependence of human health, animal health, and ecosystem health. Coming from the “One Medicine” concept (1) that advocates a combination of human medicine and veterinary medicine in response to zoonoses (2), the “One World - One Health” concept^1^ was created in 2004. The novelty was the incorporation of the ecosystem health, including that of wild fauna. The “One Health” initiative^2^ therefore constitutes a global strategy highlighting the need for an approach that is holistic and transdisciplinary and incorporates multisector expertise in dealing with the health of mankind, animals, and ecosystems (3) (Figure 2).

When one considers the multiple factors at play and the complexity of public health issues, it is clear that the “One Health” holistic approach (4) cannot be disassociated from the notion of ecological health (EcoHealth). The underlying premise is that the health and well-being of the human population will be more and more difficult to maintain on a polluted planet suffering from social or political instability and ever-diminishing resources. Supporting that view, the European ministers responsible for health and the environment as well as the World Health Organization (WHO) regional director for Europe met on June 15, 2017 in Ostrava, Czech Republic for the sixth ministerial conference on Environment and Health. They recognized that “environmental factors that could be avoided and/or eliminated cause 1.4 million deaths per year” in the WHO European Region. They declared that “public authority shares the common responsibility for safeguarding the global environment and for promoting and protecting human health for all environmental hazards across generation and in all policies.” Paving the way for ambitious integrative initiatives in the One Heath framework, researchers in “Ecohealth” and its practitioners implement systemic and integrated practices to promote sustainable ecosystemic services linked to the concept of health (human, animal, and ecosystem) and to social stability. Thus, the One Health concept provides a way of looking at complex systems and approaching processes leading to undesirable effects such as disease emergence, etc. It thus encourages and promotes the interdependence, coexistence, and evolution of living beings and their environment, which is itself in a state of constant transformation (5).

However, after just over 10 years in existence, the “One Health” concept, which predicted the integration of the interface with ecosystems in the “One Medicine” concept, has not quite completed its transformation (6). The documents and publications on the “One Health” approach, and the strategic framework developed around it, have largely focused on the battle against emerging zoonoses originating in domestic (7) or wildlife (8) and/or their interactions (9), without really considering the role of inclusive ecosystems (10). Thus, a quick review of scientific investigations claiming to adhere to the “One Health” concept clearly reveals that they only mention the environment and its biotic and abiotic components as the scene of transmission, often reduced to global planetary changes or the Anthropocene. Very few studies deal effectively with the ecology of transmission and the ecology of health meaning developing an ecological and progressive epidemiology linked to components of biodiversity, ranging from physiological stresses on populations to changes in habitat, or linked to ecosystem processes (11, 12, 13).

One of the major challenges in the successful integration of the environment alongside human and animal health in the “One Health” triptych is the capability to define the state of health of our ecosystems. Ecology researchers face a growing demand from administrators for detailed, relevant information on the health and desired equilibrium or dynamics of multifunction ecosystems to guide decision-making on sustainable development, species conservation, and human, animal, and plant health (14). This calls for the definition of shared indicators for ecosystem health (biodiversity, ecosystem services, desired “equilibrium”, and “evolutions” on relevant space–time scales, etc.).

When the “One Health” concept was conceived, initial collaboration between human medicine and veterinary medicine resulted in an inevitable research bias toward zoonotic diseases (15), temporarily ignoring the important question of chronic non-infectious diseases, which are the leading cause of global human mortality. Nowadays, the “One Health” concept hopes to extend to other fields, such as antimicrobial resistance, ecotoxicology, or health in urban environments.

In this review article, we discuss the need of incorporating ecological, evolutionary, and environmental sciences into One Health approaches for an innovative and effective control of both infectious and multifactorial non-communicable diseases. We next provide examples in which the integration of the ecosystemic component of the One Health concept enabled deciphering the processes underlying disease emergence and re-emergence. Finally, we discuss operational brakes that still limit the application of the concept, its ambitions, and future challenges.

## Infectious Diseases

### Ecosystem Dynamics and Imbalances

The emergence and re-emergence of infectious diseases are closely linked to the biology and ecology of infectious agents, their hosts, and their vectors (16). Therefore, a comprehensive understanding of ecosystem dynamics that informs on the processes leading to the occurrence or the recurrence of infectious agents, and their dissemination and extinction in natural habitats, is essential in assessing the risk of infection. The genomes of parasitic organisms, in the widest sense of the term (virus, prokaryotes, and eukaryotes), evolve in their natural environment through mutation, recombination, horizontal transfer, and hybridization. These “genetic entities” respond differently to selective environmental filters, and some genotypes are selected. These genotypes may express new phenotypes and colonize new hosts. They can also cause damage to the hosts they colonize, thereby becoming pathogens. Above and beyond the need for a comprehensive understanding of the life cycles of pathogens, transmission pathways, and transgression of species barriers, further research is required to (i) explore pathogen dynamics in natural habitats and (ii) develop models of infection close to natural systems. Developments that have been achieved for certain models, such as pathogenic vibrios in mollusks (17, 18) or pathogenic *Leptospira* in many vertebrates (19, 20), open up the possibility of a better understanding of pathogen dynamics in microbiota, interacting with a host species or a community of hosts.

Understanding ecosystem dynamics allows us to assess the degree to which the alterations caused by anthropogenic forcing lead to the development of large-scale infectious events. Historically, the domestication of animals has indirectly mediated the transfer of infectious agents between wildlife and humans (7). The majority of emerging infectious diseases considered to be significant in terms of public health also have a zoonotic origin (21), and almost three-quarters originate in wild animals (22). The study of ecological factors affecting the transmission of infectious agents in wildlife is therefore essential in understanding the mechanisms involved in transgression of species barrier (also referred to as host-switching, host-jumping, or host-shifting) and emergence in human populations. For example, the density and diversity of hosts, migration, environmental persistence, and interaction within communities of infectious agents have been identified as determining factors in the emergence of direct and vector-borne transmission agents (23, 24). Assessing the risk of the emergence of zoonoses in human populations therefore requires the analysis of interaction networks between infectious agents, their hosts, and the environment in which they evolve [for an instance of transmission of malaria between macaques and humans, see Huffman et al. (25)].

Habitat destruction and fragmentation, environmental pollution, and climate change have a confirmed catalyst effect on the occurrence and geographic distribution of infectious agents (26, 27, 28). Recent examples of epizootics, particularly destructive epidemics or zoonoses (bird flu, coronavirus, Ebola, chikungunya, dengue, and Zika) indicate that this spread was in many cases assisted by global changes. Thus, by altering the repartition of pathogens, their vectors and their reservoirs, global warming is responsible for the appearance of new diseases at northern latitudes that have previously never been affected (29–31). Particularly, noteworthy examples are the cases of schistosomiasis (32) and chikungunya emergence (33) in the European continent. The recent Ebola epidemic in Western Africa recalls that epidemics are not only limited to the circulation of viruses or knowledge of contamination principles but also strongly influenced by history, political contexts, economic inequalities, and cultural phenomena (34, 35).

In the same vein, the globalization of trade and exchange and the industrialization of agriculture, aquaculture, and agribusiness have occurred in a very short period of time when viewed on an evolutionary scale (36, 37). These trends are responsible for increased movement of humans, plants, and animals with their accompanying infectious agents, who have been able to colonize new territories. Industrialization, which has fostered intensive breeding and farming practices, has also generated stress in organisms, which in turn has created an environment that is conducive to the spread of infectious agents.

The industrialization of agriculture and farming is also responsible for the widespread and often abusive use of pesticides, fertilizers, and antibiotics, which have selected on the one hand resistance to insecticides in mosquitoes that transmit pathogens (etiological agents of malaria, arboviruses, filarioses, etc.) (38–40) and on the other hand resistance to antibiotics in bacteria (41). The selection of antibiotic-resistant strains has occurred in the same way, through abusive and poorly considered use of antibiotics in human health care. This issue now represents one of the most serious threats to global health, food security, and development for the WHO. Antimicrobial resistance is a global health crisis with multiple dimensions. Using a “One Health” approach connecting medicine with some of the well-established key concepts in eco-evolutionary dynamics is urgently needed for developing novel approaches to bacterial infection therapy for which resistance is less quick to evolve (42). Beyond research, the examples of resistance to antimicrobials and pesticides are indicative of the need to develop a policy framework that is common to public health, agriculture, and farming (43).

### Resilience, Restoration, and Eco-Inspired Control

The concept of resilience emerged in the ecological literature in 1960s and 1970s to describe the response of ecosystems to disturbances (44). In socioecology, resilience is defined as the capacity of a socio-ecosystem to absorb disturbance and to maintain particular properties such as function, structure, identity, and feedback (45). Resilience should be viewed in a dynamic way, as it allows an ecosystem to shift between different steady states, each of them possessing different sets of processes allowing functions to be maintained. On one hand, it has been advocated that an integrated One Health approach addressing the potential health effects at the human/animal/environment interface will enhance the resilience of local communities (46) through better disease prevention (47). On the other hand, the concept of resilience plaids for system-based thinking and holistic approaches, which for the “One Health” concept means to take into account the importance of diversity (from genes to species), redundancy, and adaptability of the socio-ecosystem to better face, for example, health sanitary crises.

Thus, the spread of infectious agents can be controlled by biological diversity, with predation, competition, and host–symbiont interactions, all playing a role in holobiont fitness and their dynamics, i.e., hosts and their associated microbiota. However, processes by which biodiversity can dilute or amplify disease transmission are still poorly known and are both scale- and context-dependent (48). “Demoresilience” associated with progresses in prevention and simple hygiene has not eliminated old scourges, such as plague, tuberculosis, etc., which are still infecting people and communities, but has led to a continuous decrease in epidemics, this far before vaccines and antibiotics were made available (49).

Nature can help provide viable solutions that use and deploy the properties of natural ecosystems and the services they provide. Thus, eco-inspired innovative strategies have been developed to control infectious diseases. Phages are natural predators of bacteria, controlling bacterial behavior and dynamics in the environment (50). Similarly, antimicrobial peptides, effectors of innate immunity in metazoans, and competition in prokaryotes can also influence pathogen dynamics (51, 52), vector-borne transmission (53), and may allow alternative routes of transmission in the natural environment (54). These natural control mechanisms are real sources of inspiration for the development of new anti-infectious strategies. New methods of fighting vector-borne transmission based on microbial symbiosis represent an area of research that is being promoted and encouraged on a global level by the WHO [Bourtzis et al. (55)^3^]. Likewise, just as the specter of the post-antibiotic era appears before us (56), research into alternative anti-infectives has become an international priority, once again backed by the WHO, who recommends a global action plan based on “One Health” principles (57).

We now need to evaluate the capacity of these alternatives to induce resistance and define its molecular basis in order to assess the risk. Indeed, the challenge over future years will be to identify new anti-infectious strategies likely to generate less resistance and having reduced impact on non-target organisms and the environment (58). Studies in this particular field are already underway and indicate a definite advantage to using phage cocktails (59) or antimicrobial peptides from metazoans (60) as an alternative to antibiotics. There are also promising leads opening up with the development of immunomodulatory peptides derived from antimicrobial peptides (61), whose risk of inducing resistance is extremely low.

If research is called upon to find innovative and ambitious solutions to control infectious diseases, then society, for its part, must not forget that for many extremely destructive infectious diseases, hygiene and prevention are far more effective control solutions than the use of anti-infectives or vaccines, if they exist. This also applies to various vector-borne diseases, for which education and information are the key to avoiding exposure to vectors and the pathogens they transmit.

## Multifactorial and Non-Communicable Chronic Diseases

### Toxic Risk

#### Complexity and Ambitions of Ecotoxicology

The toxic risk is implicated on many levels in the issues surrounding the “One Health” concept because of direct harmful effects of contaminants and their impact on the physiology, immune, and endocrine responses of organisms, biodiversity, and the transmission of pathogens. Contaminants and toxins can also impact host–pathogen interactions, by directly affecting the pathogens (62). However, toxins and pollutions are to a certain extent part of nature, and toxicity does not mean the same for all organisms. For example, Lake Natron (Kenya) is an unhospitable place for most species, but some have adapted to this environment (like flamingos, *Spirulina*, and invertebrates adapted to caustic waters they live on). As a consequence, the occurrence of toxicants *per se* might not be problem, and there is certainly a lot to learn from the adaptive mechanisms evolved by species living in such “toxic” environments.

Environmental pollution is *a worldwide concern*. The toxic risk is particularly high in environments where the human population is very dense, such as coastal areas, where species are subjected to multiple toxins and pollutants including natural toxins (e.g., paralytic shellfish poisoning toxins synthesized by certain harmful microalgae), emerging pollutants (e.g., micro- and nanoplastics) and diffuse pollution linked to multiple anthropogenic releases (63, 64). However, even remote areas without high anthropogenic activities such as polar areas are also contaminated, with a long list of legacy or emerging organic and inorganic compounds involved (65). The recent and global nature of environmental pollution is even reflected by marked differences in Holocene signatures in stratigraphic records showing unprecedented combinations of various anthropogenic substances (66). Wildlife and domestic animals are currently exposed to numerous contaminants at levels endangering their survival and health, their ability to reproduce and capability to cope with other stressors such as pathogens, and this represents a threat on biodiversity and ecosystem functioning which is now acknowledged (67–71).

**Figure 3:**
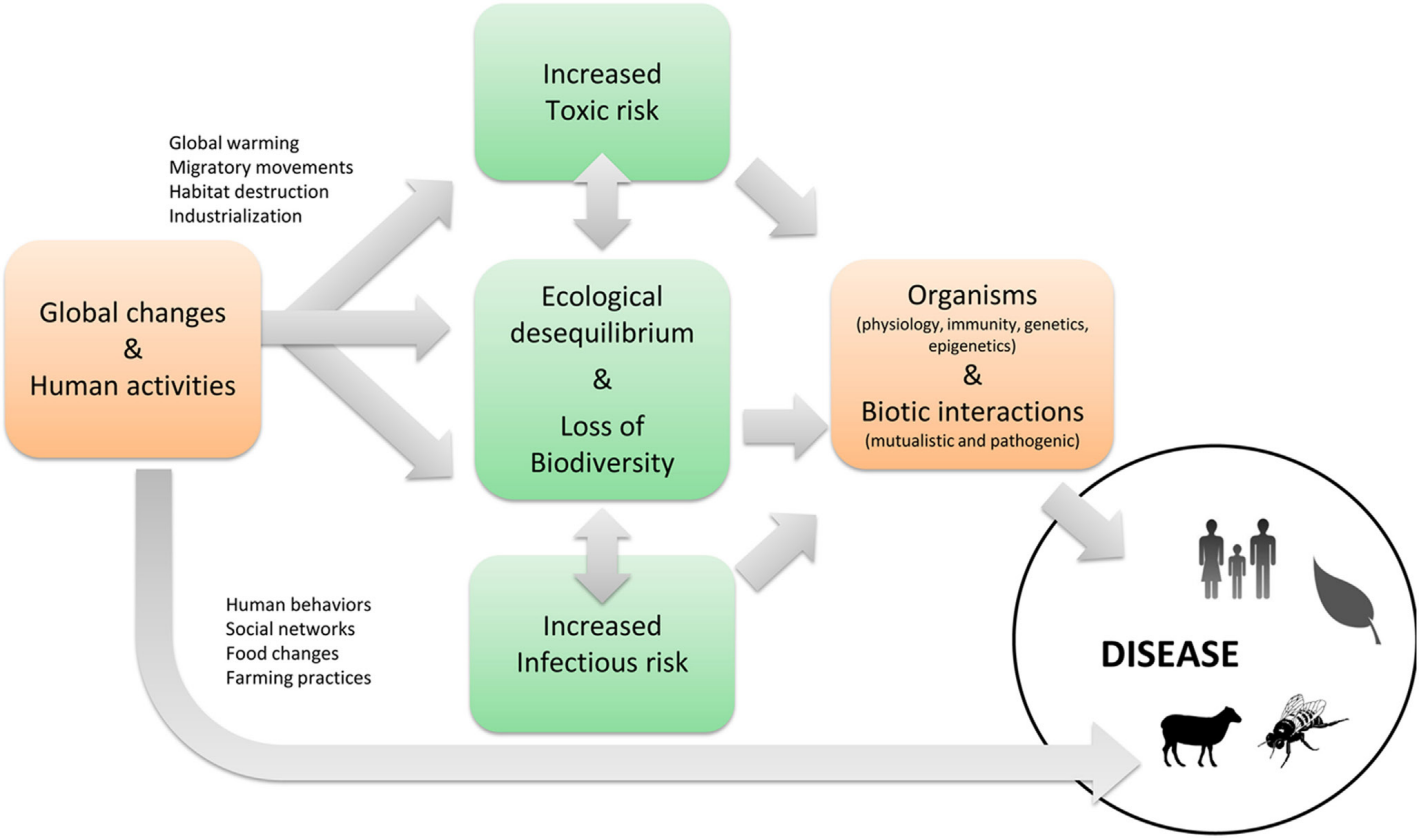
The infectious and toxic risks and their interactions.

The widescale development of *multifactorial diseases* affecting both invertebrates (bees, corals, and oysters) (72–75) and vertebrates (amphibians, cetaceans, and chiropterans) (76–79) is increasingly recognized thanks to the development of tools in genomic medicine and epidemiology that facilitate their study. As a consequence, diseases of complex etiologies are receiving increasing attention. Multifactorial diseases often emerge in organisms whose defense capacities have been reduced by changes in nutrition, temperature, salinity, pH, exposure to pollutants, toxins, radiations, etc. Through cumulative and long-term effects, toxins have significant impact on morbidity caused by both pathogens and other toxic substances (cocktails). Toxicants increase the risk of infectious diseases when the immune system is directly or indirectly affected (67, 71, 80–83). Immunotoxic effects do not only have a direct effect on human health and the viability of human and animal populations, but also affect the broader functioning of ecosystems and promote the transmission of zoonotic diseases by increasing the prevalence of pathogens in animal reservoirs or intermediary hosts. Therefore, the major threat posed by pollutants to biodiversity has currently undetermined consequences on biotic interactions (Figure 3). As a result of changes in species abundance and food web topology (extinction of “regulatory” predators, role of “super-predator,” consumptive competition, effects on keystone species, biological invasion, increase in resistant disease reservoir species, density effects dependent on emergence of epizootics or zoonotic diseases, etc.), pollution further significantly increases the risk of disease.

The occurrence of some *chronic non-communicable diseases* is currently soaring in southern countries, highlighting the globalization of sanitary risks (84). Part of it is due to significant advances in combating infectious diseases, which have greatly reduced mortality and as a consequence modified the occurrence of non-infectious diseases. However, environmental changes, and particularly exposure to toxic substances, were shown to play an important role in the occurrence of serious chronic non-infectious diseases in humans (respiratory, cardiovascular, neurological, and metabolic diseases, obesity, diabetes, and cancer), the prevention of which is a major challenge for our society, both for the present and the future generations. Transgenerational effects of environmental stress (85) transmitted by epigenetic mechanisms (86) have been described in various species. There is no reason to think that humans should be exception to this rule, and indeed a comparable picture emerges for wildlife from many case reports worldwide (70). This indicates the importance of the man–animal–ecosystem interface in determining the evolution and emergence of chronic diseases in humans, just as in other species. For this reason, human and veterinary medicine is often developing a reductionist and frequently reductive approach that needs reviewing in the context of the current situation. Prevention and control, which are increasingly accessible, have a great potential for tackling such complex disease dynamics.

#### Building a Harmonized Framework for Biological and Chemical Contaminants

Over the past two decades, a few articles called for a transdisciplinary harmonization of ecotoxicology as a component of “Ecosystem Health” and the encompassing “One Health” (81). Evidence, examples, and opportunities for cooperation have been detailed (26, 27, 81, 87–89). However, studies incorporating chemical contaminants and environmental quality in a “One Health” framework are still marginal (87). Addressing simultaneously the needs of “Ecosystem Health” and “One Health” with their inherent trade-offs is a required step forward that would undoubtedly help achieving the goal of better health for people, animals, and our environment.

Beyond integration of environmental pollution as one of the anthropogenic disturbances impairing environmental health, consideration of toxicants for their role in immunity and endocrine system would benefit from a unified framework merging the theoretical and applied contexts of eco-epidemiology, ecophysiology, and ecotoxicology (90). Pathogenic organisms and chemical pollutants have their own specificities. However, many common ecological, physiological, and biological processes rule the transmission of biological and chemical contaminants on the one hand and the exposure and responses of organisms and ecosystems on the other hand. System studies on both pathogens and toxicants not only require specialists but also joint expertise to assess impacts, manage risk, and apply therapeutic care. For instance, similar tools in mathematical modeling can be shared for trophically transmitted parasites and pollutants. This calls for more cooperation between human and veterinary medicine, functional and evolutionary ecology, institutional health-care and wildlife management, as well as socioeconomics and regulatory issues.

Furthermore, interactions between pathogenic organisms and chemical toxicants have a high interest in itself. Thus, evaluating the impacts of massive use of biocides and xenobiotics has become a priority to anticipate the consequences of such delivery on the whole ecosystem. Integrating ecotoxicological issues of biocidal substances in “One Health” should help refining the chemical control of pathogen vectors (e.g., mosquitoes) or parasites (antihelminthic, acaricide, etc.). As a first step, the development of “adaptive monitoring” approaches dealing with co-exposure to pollutants and pathogens is absolutely crucial (91–93). The challenge is to assess exposure and organism response on both an individual level and at the population level through relevant and appropriate approaches for both wildlife and human (91, 92). A further challenge consists in defining spatial and temporal scales, types of sample, biomarkers, and end points (92, 94).

#### Toward Integrated Multiscale Approaches

The ecotoxicological impact of diffuse pollution, phycotoxins, and contaminants of emerging concern, as well as their modulation by environmental factors, needs assessing today using an integrated approach that encompasses the different scales of organization of living beings (macroscopic, cellular, biochemical, and molecular) and includes studies in both controlled environments and *in situ*. The study of population response to multiple stress factors and the genetic and epigenetic bases of their capacity to adapt to these stress factors are currently priority fields of research in order to anticipate future sanitary crises that may influence the fate of species.

### Urbanization and Health

Urbanization, associated with ground and air pollution, and its role in lifestyle changes (energy-dense diets with ready-made foods that are rich in lipids, reduced physical activity, more sedentary lifestyles, etc.) represents a major environmental change for man. Since 2010, towns and cities have been the living environment of more than half of humanity (95). Our increasingly urban lifestyle leads to exposure to multiple stress factors (exposome), the health impact of which we do not yet fully understand, especially among the more fragile members of society.

The way in which people are connected and our towns constructed has an enormous impact on health, and particularly its evolution with age. A person’s social network will influence both his propensity to being infected by directly transmissible pathogens (without an intermediary host) (96) and to being affected by non-infectious diseases such as obesity (97) or blood cholesterol. However, we do not yet know exactly how urbanization, mobility, or social network nurture or hinder good health. This will require significant research. New portable detectors such as GPS or accelerometers make it possible to record extensive and precise data pertaining to people’s mobility and activity (98). The combination of this type of approach with social network measurements opens up new ways of measuring and understanding epidemics and health inequalities (96, 99). The development of statistical methods, graph theory, and multiagent simulation would make it possible to (i) identify which urban environment or social network properties influence well-being and activity and (ii) provide concrete recommendations to improve urbanization plans and public health strategies.

The notion of ecological, epidemiological, sanitary, and demographic transition seems to be a particularly federative idea in the “One Health” approach, because it allows for both the implementation of concrete interdisciplinary research (ecologists, doctors, anthropologists, biologists, demographers, etc. can work together on the changes observed) and because it is also very closely associated with the environmental change represented by urbanization, making it possible to address the subject of the etiology and prevention of chronic non-communicable diseases and infectious disease in a new and innovative manner.

## One Health Concept Successes in the Integration of its Ecosystemic Component

Some key examples illustrate the degree to which the adoption of a “One Health” approach is both consensual and particularly effective in deciphering the processes underlying the emergence and re-emergence of diseases.

### Optimizing Land Use to Control Pathogen Transmission

In 1960s, the European agriculture common policy encouraged French farmers to specialize in milk production. Farmlands from the Jura Mountains were then converted into permanent grasslands. With the destruction of hedges and increased productivity, this shifted the regional ecosystems toward large-scale small mammal pest surges with a cascade of direct and indirect consequences in agriculture, conservation, and public health, including exacerbating the transmission of *Echinococcus multilocularis*, a deadly parasite of public health concern (100). In China, similar effects came from deforestation and agriculture encroachments during 1980s (101). In such a context, research and disease regulation were necessarily considered together with the other issues. Researchers provided knowledge on ecological processes that helped stakeholders to discuss and select, in a system approach, the inherent trades off between seemingly divergent sectoral interests (102).

### Deciphering the Emergence of Infectious Diseases through Holistic and Multiple Scale Approaches

In 2013 and 2015, two independent outbreaks of Schistosomiasis occurred in southern Europe (Corsica Island, France) with around 300 estimated cases (103, 104). The occurrence of this tropical disease in higher latitude was unanticipated and caught scientists and health authorities unprepared. At the beginning of the outbreak the locals were worried, the communication was not controlled, the local physicians were not trained to diagnose this tropical disease, and the ecologists were unprepared to consider this parasite in temperate zone. Moreover, the hybrid status of the parasite, a cross between a human and an animal schistosome, made the epidemiological situation much more complex. A collaborative effort between physicians, veterinaries, biologists, ecologists, and public health institutions was set up to identify the origin of the outbreak and control it (32). The biologists identified the intermediate host implicated, defined the hybrid status of the parasite and its Senegalese origin; veterinarians proved the absence of ruminant reservoir hosts; and physicians and health authorities improved diagnostic tools, addressed the clinical characteristic of the patients, and measured the extent of the outbreak.

### Modeling Diseases in Social Networks

As the growing worldwide population becomes more mobile and urbanized the risk of epidemics is constantly increasing. Studying animal interactions and the coevolution between emerging social networks and pathogen transmission may help to predict outbreaks and develop strategies avoiding epidemics and epizootics. Network studies, especially in non-human primates, suggest not only that the position of an individual in a group affects the risk of being infected and infecting conspecifics (105) but also that the shape of interaction networks independently from individuals affects pathogen transmission (106). Over the past few years, concepts such as efficiency, resilience, and nestedness (107, 108) have been used to understand the evolution of ecological and social networks facing to environmental changes. Modeling epidemic spread in social networks should help target animals as well as humans according to their social position in the network in order to vaccine them and better manage outcome of epidemic outbreaks. Integrating ecological pressures and intra- and interspecific relationships in these models could also bring new understanding about how these networks are robust to changes and could act as buffers between the environment and animals, including humans.

Those examples illustrate how the application of the “One Health” approach to infectious risk needs to be systematically reinforced with ecobiology expertise. Similarly, the toxic risk needs to be enriched with ecotoxicology expertise. Further understanding of the risk presupposes asking a certain number of questions which may be presented in the same way for both risks (see Table 1). This knowledge of ecosystem processes must generate the signposts to guide the sustained exchange effort required from ecologists, epidemiologists, evolutionists, and human and animal health-care specialists with other activity sectors.

**Table 1:** Exploring the infectious and toxic risks through ecobiology and ecotoxicology expertise.

	Infectious risk		Toxic risk
	Case of pathogen emergence	Case of antibiotic resistance	Case of emerging toxins and recurrent toxicants
Ecosystem processes	How does a commensal infectious agent become a pathogen?	What is the ecological role of antibiotics and of their resistance genes?	What is the ecological role of toxins produced by microorganisms?

	How do infectious agents alter certain hosts?	How do antibiotics and their resistance genes operate?	How do toxins alter certain hosts?

	How do infectious agents proliferate within the microbiota of their hosts?	How do antibiotic production systems and their resistance genes proliferate?	How do organisms adapt to toxicants?

	How are infectious agents controlled in a natural environment?	How are antibiotic-producing or resistance gene-carrying populations controlled?	How are toxins controlled?

Anthropogenic alterations	How do global changes/anthropic activities impact on environments affect biodiversity and the emergence of pathogens?	How do global changes/anthropic activities impact on environments affect the emergence of antibiotic-resistant bacteria?	How do global changes/anthropic activities impact on environments affect toxic risk?

	How does the synergy between infectious and toxic factors multiply the effects and complexity of responses?		

Solutions	Which innovative control solutions are inspired by the ecobiology of infectious agents, their hosts, and their vectors?	How can we develop anti-infectives that generate less resistance?	Which control solutions are inspired by the ecology of emerging toxins?

*This table summarizes questions to be addressed for understanding the risks and develop innovative solutions*.

## Operational Brakes on the “One Health” Challenge and Recommendations

Major barriers to the effective integration of “One Health” need to be removed (i) for the systematic implementation of a “One Health” strategy and (ii) for the development of operational solutions that both respect environmental health and its future and are realistic in the face of the urgency of medical care for patients.

A major barrier to the development of “One Health” approaches is very clearly the lack of communication between human and veterinary medicine, agronomy and ecological, environmental, and evolutionary science. Removing this major impediment implies the integration of sufficient understanding of other disciplines, multidisciplinary approaches, and the aims and conditions of their implementation. This can be formulated at different levels.

From a *training* point of view, it is essential to include ecology and evolution in any medical, veterinary, and agronomic training (109, 110). Although relatively recent, a number of these training courses are currently being developed around evolutionary medicine. This initiative should be supported and strengthened in the future.

From a *research* point of view, improved *scientific cooperation* requires the development of collaborative national and international research networks (including within Europe). The integration of southern countries, with their diverse intertropical ecosystems and biodiversity hot spots, is absolutely vital as they represent genuine natural laboratories for the implementation of the “One health” concept in the face of demographic and sanitary transition resulting from global changes. Networks must also include a maximum number of key players in research, representing various disciplines and specializing in different levels of organization of living beings, and spatial and temporal scales. They must work together toward the implementation of shared training programs, tools, and protocols with a shift from research generating basic and isolated knowledge to translational research leading to systems and implicational knowledge. This certainly needs a mentality change not only from researchers but also—and even more importantly—from research funding bodies. Scientific cooperation also needs better access to knowledge, which is currently partially blocked (intellectual property, patents), thereby depriving certain key players of diagnostic criteria or fundamental knowledge (81). *Observation tools and data management programs* should also be supported. Long-term monitoring of transmission or exposure systems must be organized and supported by appropriate means and measures, including outside peaks of visible emergence, taking into consideration the different spatial and temporal scales relevant to the organisms in question (e.g., multiannual demographic variations of organisms, landscape changes, practice changes, rearrangement of communities in response to these factors, etc.). This requires the implementation of policies to collect, capitalize, secure, and make available the data derived from ambitious research (database management, observatories, etc.). In addition, promoting *multidisciplinary integrative approaches* is needed for the development of a progressive health (human and animal) ecology, which is based on acquired expertise and methodology in immunoecology and endocrine-epidemiology (111) and links the study of proximal factors (mechanisms) to their ongoing evolutionary consequences (112, 113). It is also necessary to support work into the *definition of ecosystem services* for the regulation of infectious or toxic risk (contributing to the requests of the, Intergovernmental Science-Policy Platform on Biodiversity and Ecosystem Services).

From a *political* point of view, it would appear necessary to implement a gradual *reinforcement of biosecurity* including production, transport, and transformation of biological resources. It is additionally absolutely essential to *break down the sectoral partitioning* that exists between public health, agronomy policy, and other sectors of activity (27). Indeed, current sectoral partitioning trends foster infectious risk. For instance, to date the agriculture/public health interface does not come under the competences of farmers nor vector control services, which increases the development of insect vectors resistant to agricultural insecticides. The synergy of a partnership between scientists, farmers, and the vector control services would initiate pluridisciplinary research programs whose goal would be to protect the crops while reducing the populations of vectors as much as possible. Similarly, sectoral partitioning increases the emergence and the spread of microorganisms that are antibiotic-resistant as a result of spreading slurry from farms using antibiotics or by the increase in size of host populations of pathogenic agents. It is therefore clear that a comprehensive review of industrial agriculture and farming practices is vitally urgent. Overall, significant efficacy gains can be achieved through intersectoral cooperation in a “One Health” approach. This should include a control at source, which is often more cost-effective than fighting a disease. This supposes (i) cooperation in terms of monitoring and diagnosis for a quicker and more precise diagnosis; (ii) cooperation in terms of preventative measures, such as vaccination, to increase coverage; and (iii) a detailed and immediate communication to reduce the number of cases (114).

Remarkably, a purely *economic* view also calls for a global approach of Health that relies on both “prevention at source” for animals and “control” for humans (115). It has been estimated that this two-sided approach would cost between 1.9 and 3.4 billion dollars per year to implement and optimize this approach, a sum which is far below the annual average of 6.7 billion dollars of economic losses historically suffered as a result of epidemics (114). These methods will require the consolidation of regional, national, and international approaches to biosecurity for the control of human, animal, and plant diseases and the implementation of an integrated, interdisciplinary, intersectoral approach to the monitoring of and investigation into diseases common to man and animal. A first necessary step is the development of a database that includes a corpus of essential statistics on demography, the sanitary situation, health determinants (human, animal, and ecosystem), and risk factors. These multi- and intersectoral collaborations, nourished by the results of relevant research, are also essential in identifying the bio-economically, socially, and ecologically acceptable compromises between (sometimes) contradictory management objectives (food production, health, biodiversity preservation, etc.).

In addition to prevention, ecology today must be able to offer the authorities *innovative solutions* to vector control (antivector fight) and pathogenic infectious agents (remediation) that are more ambitious and less destructive to biodiversity and ecosystems than those currently deployed. With regard to antibiotic resistance, research programs working on the identification of new anti-infectives must henceforth consider the risk of resistance emergence from the moment that new therapeutic agents are developed.

## Challenges and Ambitions for the “One Health” Concept

The insufficient consideration of certain key components in the implementation of the “One Health” concept can be highlighted. Particularly, the wildlife component and numerous related ecological issues (community ecology and evolutionary ecophysiology) are still neglected (116), as are certain environmental science components (soil and climate) (117). Additionally, social, legal, and economic sciences are similarly marginalized (118).

However, social sciences play a major role in the construction of the problems facing “One Health” research. The understanding of infectious or toxic risks cannot simply be reduced to its biological or chemical components. It is also essential to take into consideration the vulnerability, variability, and susceptibility of human societies as well as the different ways they interact with animals and ecosystems. The “One Health” concept, which promotes an interdisciplinary and intersectoral approach, must therefore engage at different levels of health governance, from a global level right down to a local level, by encouraging participative approaches that bring together communities, scientific experts, administrations, and other key players (NGOs, industry, legal experts, etc.). Infectious and toxic risks must also be addressed through their perceptions and impacts to contribute to the improvement of surveillance and prevention systems and the resilience of societies in the face of sanitary crises.

The issue of plant health as a full component of “One Health” concept is a challenge to be urgently resolved (119). In fact, human health and animal health are directly or indirectly dependent on plant health, as the latter is essential as food resources, phytomedecine, land management, etc. In terms of basic knowledge, investigations in plant ecology and epidemiology have provided useful data for understanding the mechanisms of virulence and adaptation of pathogens in humans and animals. A renowned example is the discovery by botanists of interfering RNA as a key component in gene regulation, including host–pathogen interactions (120). While some plant pathogens may pass the species barrier and cause nosocomial diseases, such as the *Burkholderia* complex bacteria responsible for human cystic fibrosis (121), others belonging to enteric bacteria (*Salmonella, Escherichia coli, Shigella*, etc.) are plant inhabitants that can cause food contaminants that are harmful to human (122). Thus, raising the concept of “One Health” to a realization requires also access to a good plant health through a productive (yield, quality, nutritional value, and biosafety) and sustainable (reducing pesticides and chemical fertilizers, encouraging culture rotation practice, biofertilization, etc.) agriculture.

The question of ethics should also be more widely integrated into the “One Health” concept. If ethics are referred to essentially through bioethics and the ethics of animal health, other components are often neglected. This is the case for environmental and biodiversity ethics, social science ethics, and the ethics of various legal concepts, such as human rights, the rights of indigenous people, environmental justice, and animal rights. The Nagoya Protocol to the Convention on Biological Diversity is one of the ethical and legal frameworks which are legally binding on scientific research, generating new consequences on the access to and sharing of microorganisms, human, animal and plant samples, data, and traditional and local knowledge and skills. Far from being a new constraint, it is an opportunity to reflect on the role of scientific research in our societies (123).

## Conclusion

This review illustrates how crucial it is to consider ecological, evolutionary, and environmental sciences in (i) understanding the emergence and re-emergence of infectious and non-communicable chronic diseases and (ii) in creating innovative control strategies. However, the actual organization of research and the sectoral allocation of resources in our societies still limit the development of transdisciplinary approaches and integrated operational actions. Removing the interdisciplinary barriers that still separate ecological, environmental, and evolutionary sciences from human and animal medicine is a major challenge to the implementation of the “One Health” concept, which moves beyond science and impacts politics (health, agriculture, aquaculture, land management, urbanism, and biological conservation), law, and ethics. There is a need to provide evidence on the added value of “One Health” approach for governments, researchers, funding bodies, and stakeholders (124). Finally, promoting the integrative benefits expected of the “One Health” concept requires a new interface with human, social, and legal sciences that remains to be built.

## Author Contributions

All authors listed have made a substantial, direct, and intellectual contribution to the work and approved it for publication.

## Conflict of Interest Statement

The authors declare that the research was conducted in the absence of any commercial or financial relationships that could be construed as a potential conflict of interest.

## Footnotes

^1^www.oneworldonehealth.org.

^2^www.onehealthinitiative.com.

^3^http://www.eliminatedengue.com/program.
